# Experiences of Vegans with General Practitioners in the Austrian Health Care System: A Qualitative Study

**DOI:** 10.3390/nu16030392

**Published:** 2024-01-29

**Authors:** Elena Jirovsky-Platter, Maria Wakolbinger, Tilman Kühn, Kathryn Hoffmann, Anita Rieder, Sandra Haider

**Affiliations:** 1 Department of Social and Preventive Medicine, Center for Public Health, Medical University of Vienna, Kinderspitalgasse 15/1, 1090 Vienna, Austria; elena.jirovsky-platter@meduniwien.ac.at (E.J.-P.); anita.rieder@meduniwien.ac.at (A.R.); 2 Center for Public Health, Medical University of Vienna, Kinderspitalgasse 15/1, 1090 Vienna, Austria; tilman.kuehn@meduniwien.ac.at; 3 Department of Nutritional Sciences, University of Vienna, Josef-Holaubek-Platz 2, 1090 Vienna, Austria; 4 Department of Primary Care Medicine, Center for Public Health, Medical University of Vienna, Kinderspitalgasse 15/1, 1090 Vienna, Austria; kathryn.hoffmann@meduniwien.ac.at

**Keywords:** vegan diet, vegan community, general practitioner, patients’ perspective, experience

## Abstract

This article explores the factors influencing the choice of general practitioners (GPs) and their role in the health care of vegans in Austria. The number of people identifying as vegan is on the rise, and GPs are increasingly confronted with vegan patients. A qualitative method was chosen for this study, and 14 semi-structured interviews with vegans were conducted between April 2022 and July 2022. Participants were recruited primarily through vegan social media groups. In their experiences with health care, vegans felt treated unequally or sometimes incorrectly. The experiences described highlight that participants felt that most GPs were biased against their veganism. Information exchange among vegans primarily takes place online and through publications of vegan associations, while GPs play a minor role in information provision. As the number of vegans grows, an appreciative way of communicating between GPs and vegan patients ought to be promoted. Voluntary interdisciplinary nutritional training, collaboration of the medical field with support organizations, provision of evidence-based information, and collaboration with dietitians and nutritionists could enrich the care of patients with a vegan diet.

## 1. Introduction

Veganism, a philosophy aimed at excluding all animal-derived products [1,2,3,4], has gained significant popularity, especially in Western societies [5]. In 2021, Euromonitor International reported that 3.4% of Europeans followed a vegan diet [6]. In Austria, the number of people defining themselves as vegan has also increased [7,8,9], prompting the development of vegan dietary recommendations [3,10,11].

The beneficial effects of a vegan diet have been shown by several meta-analyses [1,12,13], which have revealed that individuals following a vegan diet have significantly lower BMI, blood lipids, and fasting glucose values compared to omnivores. Prospective cohort studies have also suggested a lower risk for ischemic heart disease [14,15]. However, a vegan diet is not automatically associated with positive effects, as the degree of food processing must be taken into account [16,17,18]. There are also studies raising concerns about potential adverse effects, as vegans have lower bone mineral density and an increased risk of fractures compared to individuals on a mixed diet [19]. Furthermore, data suggest that a plant-based diet, particularly a vegan diet, may be associated with lower grip strength and lean muscle mass [20]. While several international scientific societies have stated that a well-balanced vegan diet is not necessarily associated with risks of nutrient deficiencies, vitamin B12, vitamin D, zinc, iron, and iodine may be critical nutrients [21].

In Austria, the healthcare system is based on compulsory health insurance, and general practitioners (GPs) are recommended as the first point of contact. However, patients are free to contact a specialist in the ambulatory care sector directly, and they do so in high numbers [22,23]. This allows individuals to choose their preferred GP. For GPs with a health insurance contract, patients do not have to pay. There are a few exceptions (e.g., self-employed persons, persons in the public sector) who have to pay a deductible [24]. In addition, there are also GPs without health insurance contracts for whom you have to pay and who are partially reimbursed by the compulsory insurance [25].

GPs are particularly crucial in addressing lifestyle-related questions because they typically have more frequent and regular interactions with patients [26]. Previous studies in the general population have demonstrated that patients attribute a high level of competence to GPs regarding nutritional matters [27]. On the other hand, medical students and residents report less nutrition-specific training [25,28], calling for more emphasis in medical school curricula worldwide [29]. A French study also highlighted these knowledge gaps, finding that 50% of physicians preferred to adapt their routine patient care to individual dietary choices [30].

Health literacy plays a relevant role in the acquisition of health information. The Healthy People Initiative defines health literacy as the ability to “find, understand, and use information and services to inform health-related decisions and actions for themselves and others” [31]. Lower health literacy is correlated with increased mortality and morbidity and lower adherence [32]. Sources of health-related information, including nutritional information, are diverse, ranging from newspaper articles to friends and acquaintances, the Internet, and e-health applications [33,34]. A systematic review addressed the high prevalence of misinformation on social media [35].

There are many motivations for becoming vegan. A 2008 qualitative study in the United States, Canada, and the United Kingdom identified the most important ones [36]. The two main reasons for omitting meat are ethical and health reasons. The researchers also demonstrated that for many people, the initial motivational reason changed over time; for example, for vegetarians who were initially motivated by ethics alone, health aspects became increasingly important. Other studies have yielded similar results, with ethical reasons—not killing animals—consistently being the primary motivating factor [37,38]. Although ethical and health concerns were at the forefront of reasons for becoming vegan, a 2015 study highlighted that vegans and vegetarians face many societal stereotypes, prejudices, and negative emotions directed against them [39]. For example, in the early 2010s, newspapers in the United Kingdom portrayed veganism in a derogatory and ridiculed way, and vegans were framed as ascetics at best but also as extremists [40]. The term “vegaphobia” was coined to describe the derogatory discourses about veganism and the social aversion to vegans and vegetarians [40].

Stigma has been defined as an “attribute that is deeply discrediting”, which may include the avoidance, shaming, or ridicule of individuals with this attribute [41]. Existing literature suggests that vegans are vulnerable to such stigma because of their “dietary deviance” [42]; they “disrupt social conventions related to food” and, in particular, meat consumption [5,40,43]. Vegan stigma does not prevent people from adopting a vegan lifestyle [38].

How vegans experience the Austrian health care system, where they obtain their health-related information, and what role their GPs play have never been studied. Therefore, we investigated the factors involved in choosing a GP and wanted to determine what role GPs play in the health care of vegans (e.g., how competent vegans consider their GP to be in providing vegan-specific information and what it means to them to be a vegan patient in the Austrian health care system). We wanted to understand where vegans in Austria obtain their information about vegan-specific health care.

## 2. Materials and Methods

A qualitative study design was chosen for this study. Veganism is an under-researched socio-cultural phenomenon closely linked to personal lifestyles, life worlds, and social groups, and a qualitative design allows individual perspectives to be explored in-depth [20]. Qualitative methods are widely applied in health research, as they are well suited to understanding patients’ lived realities [44]. This study aims to report according to COREQ guidelines [20].

This qualitative study involved semi-structured interviews with 14 vegans in Austria. It was conducted by a male medical student living vegan for his medical thesis under the supervision of S.H. and M.W [45]. All other authors are not vegan. Interviews took place between April 2022 and July 2022.

Individuals over the age of 18, living in Austria, and who had been vegan for at least six months before the interview were recruited via postings in veganism-related Facebook groups and public bulletin boards. Facebook groups with a general focus on veganism were chosen, as we assumed that groups with a particular health-, activist-, or ethical focus towards veganism would have distorted the outcome of our study, especially in terms of motivation for the vegan lifestyle. Furthermore, a snowballing method was applied [46]. Since public postings were made for recruitment, it cannot be determined how many people refused to participate, for what reasons, or their particular characteristics. People who were temporarily or permanently incompetent were excluded. All participants signed informed consent forms, and the Medical University of Vienna Ethics Committee granted ethical approval for this study (1123/2022).

An attempt has been made to select people with diverse demographic backgrounds in terms of age, highest level of education, and gender. However, this was rather difficult in practice, as most people contacted to participate corresponded to a specific demographic background: female, young, and educated. However, since it cannot be assumed that all vegans in Austria are homogeneously distributed across all demographic strata, this selection was retained. Instead of meeting the representativeness criterion, which plays a more significant role in quantitative studies, care was taken to achieve data saturation [47]. No new observations or insights were gained after 14 interviews.

Interviews were conducted in quiet surroundings of the participant’s choice. Three interviews were conducted via Zoom due to the restrictions of the COVID-19 pandemic. All interviews were audio-recorded and transcribed verbatim. For better comprehensibility, idioms were translated into standard language. All interviews were analyzed inductively and deductively [48] using the software MAXQDA (MAXQDA2022) by the interviewer and E.J.-P.

## 3. Results

### 3.1. Participant Characteristics

Fourteen individuals who had been vegan for at least six months participated in the study (Table 1). Of these, eight were female, three were male, and three identified as non-binary. In the following results section, the gender pronoun “they” is used when referring to one of the latter. The age of the participants ranged from 22 to 62 years, with the majority being middle-aged. Educational levels were very homogenous, and all but one were employed at the time of the interviews.

### 3.2. Motivational Reasons—Veganism and Perceptions of Personal Health

For the study participants, the vegan diet was a crucial part of their lives and gave them a sense of identity. They considered it to be more than just a diet. The main reasons for switching to a vegan diet were animal ethics and environmental concerns. One female participant stated that she had initially changed her diet to lose weight. None of the study participants mentioned health reasons as a decisive factor in the change. However, some reported that over time, and after looking more closely at veganism, the health aspects of veganism had come to the fore.

The most frequently mentioned aspect of how a vegan diet affects health is a more conscious approach to food. A vegan diet involves a limited choice of foods, so vegans need to acquire extensive knowledge about alternatives, which often leads them to choose what they consider to be the healthier alternative. According to the participants, vegans also need to learn about the nutrients required for a vegan diet, which increases their health awareness. All of these aspects led to a perceived healthier diet, even among participants whose motivation for the vegan lifestyle was not health-related. A 26-year-old female participant (A04) mentioned that through her dietary changes, she had discovered healthy foods that she was unfamiliar with. Another participant (C03, 30 years old, non-binary) said that they eat vegetables and fruits more often. They also considered vegan products to be generally healthier than animal products—even if these vegan products were “unhealthy” food options.

In addition to the perceived higher nutritional value of vegan foods, veganism was described as having a positive effect on mental well-being due to a better conscience when consuming vegan foods.

### 3.3. Health Care Experiences of Vegan People

#### 3.3.1. How Vegans Want Their Physicians to Be

In general, in order to trust their physicians, study participants wanted to be taken seriously, for the physician to be non-judgmental about their veganism, for them to be listened to, and for there to be general sympathy and empathy. One non-binary participant described it as follows:

*And the other thing is that it’s really important to me that I’m taken seriously. So that the person somehow responds to me, listens to me, takes what I say seriously, is somehow also a little empathetic—doesn’t have to be fully empathetic, but at least gives me the feeling, ok, the person believes me, what I say and goes into it and takes me seriously and perceives me as a person and not just as a file in the computer or something. (C02, 34 y, non-binary)*

It was also crucial for physicians to consider, or at least include, patients’ perspectives on the causes of their illnesses in their diagnoses. One participant in particular, a 30-year-old non-binary person, wished for more bidirectional communication in this regard:

*I would say [the second most important thing] for me is that I’m taken seriously and not [dismissed]. So, if I get there with my own [ideas], with something that I know for myself or think I know, that [the reaction is not:] “Ah yes, Dr. Google” and [that I am] not taken seriously, but simply [that] what I come up with, is taken up. I think that’s really important, in both directions, that if I really say something that is complete nonsense… or believe that it will be explained to me in an appreciative way… but [I wish] that it’s simply possible, that there’s a [place for me] to say “OK, I have the feeling that maybe it could be this or that” like that. (C03, 30 y, non-binary)*

The concern was that the physician would dismiss them as misinformed patients whose views were not worth considering. Similarly, a neutral or even positive perception of veganism was considered very important, as a 43-year-old female participant mentioned:

*[…] so if I have someone who already has a rather negative attitude towards veganism, then I can’t build up any trust or anything like that because I always have that person in the back of my mind who is judging me or him, I don’t know how to say this, it’s just difficult, you just don’t have as good a feeling as with someone who you know is pro veganism and supports you on your journey. (A01, 43 y, female)*

The same participant reported switching to a physician who specifically advertised to be “vegan-friendly”:

*…and then, yes, I was almost a little bit flashed, in the beginning, when I was with the new doctor, to be treated so unprejudiced and how much trust that created, that wasn’t explicitly a positive thing, but that was just a good start to this doctor-patient relationship (A01, 43 y, female)*

The experience of having a vegan-friendly physician was eye-opening and led participants to trust them more. The feeling of being singled out as a “special case” in a world of omnivorous patients was less with a physician who “already knows the ropes” about veganism (A07).

#### 3.3.2. Physicians’ Knowledge about the Vegan Diet

The common denominator among the study participants was that they wished their physicians had basic competence in veganism and vegan nutrition. Only one of the fourteen participants considered their physician’s knowledge irrelevant because veganism had been an ethical choice and was not conducted for health reasons. For the others, basic competence was necessary because they wanted to ensure that their physicians would recognize potential (threatening) health problems directly related to veganism, such as nutritional issues or blood values. A 30-year-old non-binary person explains:

*It always depends on what kind of problem I have. Because, if I really want to have my blood count checked, then I would like to talk to someone right from the start who also has a plan for it. (…) If it’s just about standard little things like a cold, or a sore throat, or an earache, then it’s not that important to me. (C01, 30 y, non-binary)*

They continued that competence in veganism is also needed for other specific reasons, such as vegan medication.

*What if, for example, my doctor, my general practitioner, tells me yes, okay, you need the medication now, but then immediately says, unfortunately, there is only that [non-vegan option] available at the moment; it’s not available in vegan, and there are no alternatives. And if it’s something like that, even if it’s just a little bit, you notice that there’s a little bit of knowledge there, too. (C01, 30 y, non-binary)*

On the one hand, it would be optimal for vegans to have a vegan medication available. On the other hand, the participants felt that if a physician addressed the issue, it would show at least some awareness of the unique needs of vegan patients and some respect for their dietary choices. Some physicians who were aware of the participant’s vegan lifestyle still prescribed medication containing animal products. For instance, C02 only found out that the prescribed medication was not vegan by researching it on the internet. It led them to not pick it up from the pharmacy, and they had a strong feeling of not being taken seriously.

Participants wished that their physicians were trained in healthy vegan nutrition; some even felt entitled to demand that they be trained in vegan nutrition. For example, one female participant in her late twenties mentioned her standards:

*[…] I have high standards for it because I know that not all people have that much time to find out more or even know where they can look for certain information. And in that respect, it would actually be the job of the general practitioners. Yes, they are the doctors and not the patients, so in that respect, I would have a social claim (laughs). (A07, 29 y, female)*

She considered it a physician’s social responsibility to gain knowledge about veganism since it cannot be assumed that all vegans have the same resources to obtain the necessary information.

Another participant considered it preferable for the physician to address the need for further research before passing on incorrect information.

*[…] it would be important to me that with my family doctor and other family doctors, it would be the case that the person also admits that it is not their area of expertise and then refers them to some experts, in other words, really saying “if you have questions about nutrition, take a look. I have a few business cards from dieticians, or I can refer you to a well-known nutritional doctor”, for example. So, for me, it’s basically the case that even a doctor doesn’t have to and can’t know everything, but it’s their responsibility to refer things to an expert. (A04, 26 y, female)*

It was considered essential and trust-building for physicians to be open and honest if they were unfamiliar with veganism or veganism in general, and then to research it if necessary.

#### 3.3.3. Prejudice against Vegans

Several study participants said they had not told their physicians about their vegan diet. Some feared being judged, others wanted to avoid discussions, especially when visiting a new physician, or feared that they might advise them against veganism, as the following two quotes illustrate:

*[That is what] I see as a danger that problems might then be blamed on the diet, which with Omnis might not be blamed on the diet. Well, my family doctor knows you’re vegan, you have this and that problem, and then they might say, “Okay, that’s because you’re vegan, because you’re vegan”. That’s perhaps also a reason why they don’t do this to themselves or why they don’t come out [as vegans]. (B03, 27 years, male)*

*A big difference for me would be in myself, that it’s always been that way since then, i.e., I have a few chronic illnesses and I’m always afraid that they might think that it’s because I’m vegan. Because it’s always a big topic for me, especially when I’m with new doctors or something like that, I usually don’t want them to find out at the beginning that I’m vegan. So that they don’t say, “Ah yes, okay, that’s why”. (C03, 30 years, non-binary)*

It was felt that physicians would immediately attribute any reported symptom to the vegan diet. Participants feared that this would ultimately lead to less diagnosis of their health problems and that the only recommendation they would receive would be to go back to eating animal products. There was a palpable uncertainty that more serious illnesses might be overlooked. Specific incidents, such as the following, were described:

*[…] A friend of mine had an underactive thyroid and went to the doctor with it, and the doctor refused to send him further or have him examined because he said, “No, all the symptoms don’t need it; it’s definitely all about veganism”. Exactly, and then that’s why it was almost, well, it wasn’t discovered for a very long time. Only later, when he made an appointment for himself again. (C03, 30 y, non-binary)*

Another participant reported that a friend had started consuming animal products again on the advice of a physician.

*[…] headaches were somehow blamed on veganism. The person really had a chronic headache and actually ate the recommended foods again against their will—it didn’t help. (B02, 22 y, male)*

It seems that, in this participant’s view, the aforementioned friend was mistreated and coerced into changing his ethically motivated vegan diet without any positive outcome.

### 3.4. Vegans’ Management of Health-Care Related Difficulties

#### 3.4.1. Information Acquisition in the Vegan Community

The Internet plays a significant role in the exchange of information about GPs and, thus, in the choice of a physician. On the one hand, social media platforms, especially Facebook, were frequently mentioned, and specific websites, such as the website of the “Vegan Society of Austria”, published a “list of vegan and vegan-friendly physicians”. Several participants reported finding their physicians on this particular list.

Social media platforms were generally seen as an opportunity to share ideas and experiences, as vegans often do not have an exclusively vegan social circle. Facebook groups were seen as having the advantage of bringing many vegans together in one place.

*I think this is an opportunity that a lot of people use to exchange ideas and get experiences, testimonials, or tips, um, because not every person in your environment has 20 vegans, and you have them there all together and can gain a lot of knowledge, get tips or even share your own experiences. That plays a very big role. (C02, 34 y, non-binary)*

Participant B03 described how people specifically search for vegan physicians in Facebook groups:

*Exactly, so I know it’s typical that in vegan groups, you read, “Does anyone know a vegan, a vegan xy doctor?” I definitely hear it very often from pediatricians, but also from family doctors. (B03, 27 y, male)*

Participant C03 stressed that testimonials on the Internet played a greater role in their choice of specialists than in their choice of a GP, as they felt that there were not enough GPs conveniently located nearby. For a specialist, they were prepared to travel a longer distance.

*[…] it’s a much bigger one with specialists because I’m willing to travel further for them, and then when I hear that there’s a doctor and they also have a vegan focus or are vegan themselves or have already had one with someone reacted well. Then that will more likely influence my decision. Much less with general practitioners, because there are simply far too few positive experiences, so if at all I read “it was bad there, it was bad there, it was bad there”, then good in between, but not now, there is none. There are enough positive reviews that there is a good chance that there is someone in my area. (C03, 30 y, non-binary)*

It seems that it did not make sense for this participant to change the physician and look for a vegan-friendly alternative, as GPs in general do not have a good reputation in the vegan community in Austria. This is in contrast to medical specialists, where more positive experiences were shared within the vegan community.

#### 3.4.2. Motivations for Changing the Physician

Seven of the fourteen participants stated that they had changed physicians, or at least considered it, because of their vegan lifestyle or their physician’s reaction to their veganism. For those participants who had considered a change, practical reasons, mainly distance, were the main reasons for not following through.

Regardless of wishes for the characteristics of the specific treating physician, practical aspects, especially proximity, played a significant role in the choice of physicians. Traveling very long distances for consultations, usually for minor problems such as a cold, was not considered acceptable. Participants were willing to compromise on other aspects, such as competence in veganism, if their GP was conveniently close to their home address, as the following remark illustrates:

*It would be very important to me [competency on veganism], and I always think about whether I shouldn’t change, just the doctor that I know or the few doctors that are far away from where I live, and that is, I actually only go to the doctor, so to speak, when I’m really not feeling well, and then the idea is that I’ll have to drive three-quarters of an hour through the city (A06, 39 y, female)*

Other participants changed their GPs despite the inconvenience of the new GP being in a different district. For instance, participant A07 changed physicians because of an experience in which her previous one mistakenly interpreted a high level of homocysteine as positive when it indicated a possible vitamin B12 deficiency. As a result, her confidence in his competence suffered greatly, and she switched to a physician who expressly declared himself vegan.

*Well, I mainly switched away from that in the 14th, in truth in some form, because of the diet, because I simply felt that I was in such a bad place there, because of this homocysteine misinterpretation moment and, so to speak, I actually already did in some form decided for him now because of his own vegan lifestyle, exactly, yes. (A07, 29 y, female)*

Participant A01 reported that she changed physicians because her previous one blamed her veganism for all her ailments. She feared that certain conditions might be overlooked:

*The consequence of this whole long odyssey with this doctor was that at some point, I looked for another doctor so that I wouldn’t have to be upset about it anymore and no longer have to think that no matter what I have, it is Eh, veganism—what if I have something [serious], then he’ll say it’s because I’m vegan. Then maybe he won’t even investigate it. (A01, 43 y, female)*

Experiences of being ridiculed were also reasons for changing physicians. At the first visit, participant C03′s physician made comments that were unacceptable to them:

*I don’t remember exactly, but it was just a bad joke about how he’ll see me more often now that I’m vegan or something like that. That got on my nerves so much that I said “no” (laughs). (C03, 30 y, non-binary)*

This last quote again refers to the many prejudices that exist: veganism seems to be considered unhealthy, and vegan patients are not taken seriously.

## 4. Discussion

This article explores vegans’ experiences and wishes with GPs in Austrian health care. It shows the desire of vegans for competence and impartiality regarding their ethically motivated nutritional choices. The desired characteristics, such as listening, valuing the patient, impartiality, and attention to prevention, are common wishes. They are not specific to vegan patients. The article also shows that, from the accounts of the vegan participants, they felt that there are prejudices against veganism and that they were stigmatized. Furthermore, it portrays how vegans try to navigate the healthcare system based on information from social media to avoid further negative experiences.

### 4.1. Feelings of Stigmatization

Published studies have described stigmatization of vegans [5,34,36,37,38], and our results resemble these previous findings: the study participants felt stigmatized, felt ridiculed as being more prone to sickness, and assumed that their physicians explained any of their symptoms with their veganism without further medical investigation. The participants considered it negative that physicians recommended returning to consuming animal products based on their personal opinion rather than on medical evidence. It should also be noted that this is the subjective point of view of vegans and that the opinion of GPs is not known here. Additionally, it is not known in what context the GPs suggested these dietary changes. The participants feared that serious illnesses might be overlooked simply because they had made an ethical choice that they felt was healthy and morally right.

These findings regarding vegan patients resemble findings on biases against individuals with obesity documented in the health care setting. A scoping review showed that weight bias by primary healthcare professionals negatively affects patient engagement with primary health care services [49]. A qualitative study displayed that patients with obesity develop negative attitudes toward future healthcare encounters, leading to an escalation of unhealthy behaviors [50]. Individuals affected by weight stigma can suffer from physical and psychological harm; weight bias also negatively influences public health policies, access to care, and research. For this reason, a consensus statement on ending obesity stigma was published in Nature Medicine in 2020 [51]. We argue that the same points that should be made against weight stigma also apply to vegans.

Considering that the majority of our participants were female, a reason for the experiences of not being taken seriously could also point to an already well-described gender bias in the clinical management of various health problems [52,53,54,55,56,57,58,59].

### 4.2. Physicians’ Knowledge

This study highlights the importance of more interdisciplinary collaboration between health professionals and the inclusion of nutrition-specific education in medical training. The results are similar to those of Villette et al., showing gaps in knowledge about a vegan lifestyle among GPs [30]. It also confirms other studies. A mixed-methods study from the US published in 2021 asked medical students and physicians-in-training in general medicine how knowledgeable they thought they were about general and vegan-specific nutrition topics, and only 22% stated receiving sufficient training in this area during their studies [60]. A qualitative interview study published in 2017 and a systematic review from 2019 also emphasized the need for a greater focus on nutrition in medical studies at universities [28,29].

A search for the keyword “nutrition” in the course catalog of the Medical University of Vienna in 2022/2023 yielded 7 nutrition-related elective courses, which are not compulsory. A compulsory course block in the 5th semester of 20 working days is entitled “Nutrition and Digestion”. Individual lecture units dealing with nutrition can also be found in two other course blocks [45]. However, it is impossible to determine whether veganism is ever addressed since it is such a small topic. The question arises: How much do physicians need to know about nutrition? Would interdisciplinary collaboration between physicians and nutritionists be sufficient? Would it be enough to refer vegan patients to them? What could be practical solutions to this issue?

On the one hand, limited nutritional knowledge is not a problem specific to veganism, as physicians generally have little nutritional knowledge about food intolerances and celiac disease [61]. On the other hand, nutrition is an integral part of prevention, and physicians in Austria are not compensated for prevention or taking extra time [62]. We do not believe that every physician needs to specialize in every area of nutrition, and it would be good if patients did not assume that physicians are familiar with every specialized form of nutrition. Physicians cannot know everything but must know where their knowledge ends and refer patients to the right place. Thus, patients should be informed that dietitians are more specialized in such specific nutritional issues. However, in Austria, the costs of dietitians are only covered in hospitals, rehabilitation clinics, and specialized outpatient clinics, but not in primary care [63]. A possible solution could be to improve interdisciplinary collaboration with dietitians and nutritionists.

In addition, voluntary interdisciplinary training could be offered as the number of vegans continues to grow. This training could also include training on how to deal with vegan patients in a more informed way. We will discuss these points further below. Currently, an initial assessment tool is also being developed to support physicians in caring for vegan patients, which can also be used by vegans themselves: https://www.veganscreener.eu/ (accessed on 23 January 2024). Additionally, vegan food pyramids, such as the Gießen Vegan Food Pyramid [64], or different fact sheets created by the various nutrition societies can be used to educate patients [11,65].

### 4.3. Vegan Medication

One of our participants reported that he was given a medication based on animal products and decided not to take it—a decision that could potentially be hazardous for his health. The issue of medication concerns vegans, allergic people, individuals with food intolerances, and patients with strict religious dietary regulations. A systematic review from 2021 concluded that more insight is needed into how religious or cultural beliefs affect the acceptance of animal-derived medications and that physicians need to be aware of animal-derived ingredients and what is appropriate for their patients [66]. In Austria, physicians do not see in the medical software whether a medicine is animal-derived, lactose-free, etc. It would be a far-reaching recommendation concerning many different groups of people to have automated alerts or easy access to this information by default. For a physician, it is not feasible to remember all medications and generics, including all variants for all types of intolerances/vegetarianism. Pharmacies could also assume a certain responsibility for seeking a fitting alternatively formulated medication/generic if a patient asks for it.

### 4.4. Health Information Acquisition

Health literacy, among others, is a prerequisite for equitable health care, which must also be emphasized concerning vegan patients, although the majority of them are well-educated [16,67]. Research has also indicated that vegan people have a solid understanding of general nutrition and are likely to have high levels of health literacy [68,69]. However, if people do not feel they can trust their physicians to be well informed about their issues and rely on social media as their primary source of health information, this might negatively affect their health care.

The results suggest that GPs in Austria play a minor role in providing vegan-specific knowledge, as vegans consider their knowledge to be insufficient. Vegans in Austria rely on information provided, for instance, by the Vegan Society and the list of vegan-friendly physicians. This in itself can be seen as proactive self-help. However, the fact that the Internet, friends, and acquaintances are the primary sources of information is a cause for concern because, on the one hand, it might be difficult for vegans to assess which sources are trustworthy. On the other hand, healthcare providers do not know how good the information available to their vegan patients is, i.e., how much correct information they have. It has been established that there is a high prevalence of misinformation on the internet [35]. As the internet plays such a dominant role in information acquisition, one solution for this dilemma may be to systematically evaluate online content to establish a baseline for the quality of the information provided. Furthermore, evidence-based information on vegan nutrition should be presented in a high-quality and straightforward manner so that vegans can find the correct information about their nutritional needs. Collaboration between medical universities, research facilities, and support organizations must be encouraged to provide vegans with the best information available to ensure equitable health care. In this context, it should be mentioned that very restrictive variations of a vegan diet, such as fruitarianism, in which mainly fruit is eaten in combination with nuts and seeds [70], are promoted on the net as well, although a purely fruit-based diet can lead to malnutrition.

One must also note that there is a growing market for vegan convenience food and that these products are often sold as particularly healthy. It is difficult for consumers to tell the difference between what is actually healthy and what is sold to them as healthy. Some of the widely available products are ultra-processed foods (UPFs). It has been shown that not every vegan food is per se healthy [16,18] and that there are potential adverse effects of vegan or vegetarian UPFs on health [17]. These facts also need to be made widely known to vegans.

### 4.5. Vegan Culture

A 2006 relational study concluded that veganism is a “diffuse” cultural movement in which social networks play an enormous role in sustaining a vegan lifestyle and thus the vegan movement as such; it is not a solitary practice [71]. Furthermore, veganism is context-specific [5] and subject to intersecting social dynamics such as gender, ethnicity, sexuality, or religion [72]. It differs from the dominant “meat culture”, which refers to the “representations and discourses, practices and behaviors, diet, and tastes that generate shared beliefs about, perspectives on, and experiences of meat” [73]. As mentioned above, this “abnormality or deviance” of veganism, for instance, leads to “vegaphobic” discourses and a notion of “deviant” vegan others [40]. Usually, being “the other” also leads to even stronger social bonds among these “others”.

Having established the “cultural nature” of veganism, cultural competence, and cultural humility come into play in healthcare. Healthcare professionals need to be aware of their patient’s cultural beliefs and practices and acknowledge that their patients’ views may differ from their own. It is also important to recognize that an individual’s self-identification with cultural norms and expressions may change over time and depend on the individual’s life experiences and circumstances [74,75,76]. Overall, miscommunication due to ignoring culture leads to inequity in health care [74,75,76]. This also applies to nutrition: knowing a patient’s dietary habits is essential to providing quality and equitable health care [77,78].

Ideally, physicians should be open to reflecting on their own biases and be willing to take the lead in learning about the unique needs of their patients. However, physicians also need enough time to care for a diverse patient population, which they often lack in the systemic conditions of the Austrian public health care system, which fosters brief encounters between patients and physicians.

Taking a vegan patient history could be guided by screening questionnaires for cultural explanatory models [74], cross-cultural interview questions about end-of-life care wishes [59], or questionnaires to understand the structural circumstances of a patient’s life [79,80], among many others. The most critical question to start a conversation is: “How can we optimally work together to ensure that you remain healthy and receive optimal care as a vegan—or better yet—as a human being with different specific expressions, diseases, and other peculiarities?”

### 4.6. Strengths and Limitations

This paper has significant strengths in that it provides a thorough, in-depth analysis based on the personal experiences of vegans, thereby offering a comprehensive understanding of the issue. Furthermore, we ensured data triangulation and strove for data saturation to ensure the credibility and validity of the findings [47]. Nonetheless, this study encounters specific limitations. It lacks the perspective of GPs, which would be very relevant to getting a holistic picture. In response, an ongoing online survey aims to gather insights into the attitudes and knowledge of Austrian physicians regarding veganism. Furthermore, generalization poses a significant concern, as the findings do not universally apply to the entire vegan community. Moreover, potential bias and limited representation of diverse perspectives may arise from the sample size and homogeneity, characterized by a predominantly well-educated and female population. It is crucial to recognize the likelihood of selection bias, given that participants were primarily recruited through social media channels. In the future, a more extensive survey based on these qualitative findings would significantly enhance the robustness of the conclusions.

## 5. Conclusions

This article examines the experiences of vegans with GPs in the Austrian healthcare system. The results suggest that GPs in Austria play a minor role in providing vegan-specific knowledge. We have highlighted that participants felt that most GPs were biased against their veganism, which could be seen as similar to the weight bias against patients with obesity in healthcare settings. As the number of vegans grows, an appreciative way of communicating between GPs and vegan patients ought to be promoted. Patients need to be informed that GPs generally are not the first point of contact for nutrition-specific questions but that dietitians are more specialized in specific nutritional issues. Voluntary interdisciplinary training, the collaboration of the medical field with vegan support organizations, the provision of evidence-based information, and the use of available (vegan-specific) tools are needed.

## Figures and Tables

**Table 1:** Descriptive Characteristics of Interview Participants.

	Self-Described Gender	Age	Completed Education	Employment Status
A01	Female	43	Apprenticeship certificate	Employed
A02	Female	41	Higher education	Employed
A03	Female	35	High school diploma	Employed
A04	Female	26	Higher education	Employed
A05	Female	57	High school diploma	Self-employed
A06	Female	39	Higher education	Employed
A07	Female	29	Higher education	Employed
A08	Female	62	Higher education	Employed
B01	Male	31	Higher education	Employed
B02	Male	22	High school diploma	Employed
B03	Male	27	High school diploma	Employed
C01	Non-binary	30	High school diploma	Employed
C02	Non-binary	34	Higher education	Unemployed
C03	Non-binary	30	Higher education	Employed

## Data Availability

The data presented in this study are available on request from the corresponding author.
